# Clinical and neuropathological features of ALS/FTD with *TIA1* mutations

**DOI:** 10.1186/s40478-017-0493-x

**Published:** 2017-12-07

**Authors:** Veronica Hirsch-Reinshagen, Cyril Pottier, Alexandra M. Nicholson, Matt Baker, Ging-Yuek R. Hsiung, Charles Krieger, Pheth Sengdy, Kevin B. Boylan, Dennis W. Dickson, Marsel Mesulam, Sandra Weintraub, Eileen Bigio, Lorne Zinman, Julia Keith, Ekaterina Rogaeva, Sasha A. Zivkovic, David Lacomis, J. Paul Taylor, Rosa Rademakers, Ian R. A. Mackenzie

**Affiliations:** 1 Department of Pathology and Laboratory Medicine, University of British Colombia, British Columbia, Canada; 2 Department of Neuroscience, Mayo Clinic Jacksonville, Jacksonville, FL USA; 3 Division of Neurology, University of British Columbia, Vancouver, BC Canada; 4 Department of Biomedical Physiology & Kinesiology, Simon Fraser University, Burnaby, BC V5A 1S6 Canada; 5 Department of Neurology, Northwestern University Feinberg School of Medicine, Chicago, IL USA; 6 Department of Pathology, Northwestern University Feinberg School of Medicine, Chicago, IL USA; 7 Division of Neurology, Sunnybrook Health Sciences Centre, Toronto, ON Canada; 8 Department of Anatomic Pathology, Sunnybrook Health Sciences Centre, Toronto, ON Canada; 9 Tanz Centre for Research in Neurodegenerative Diseases, University of Toronto, Toronto, ON Canada; 10 Department of Neurology, University of Pittsburgh School of Medicine, Pittsburgh, PA USA; 11 Department of Pathology, University of Pittsburgh School of Medicine, Pittsburgh, PA USA; 12 Department of Cell and Molecular Biology, St. Jude Children’s Research Hospital, Memphis, USA; 13 Tennessee and Howard Hughes Medical Institute, Chevy Chase, MD USA; 14 Department of Pathology, Vancouver General Hospital and Vancouver Coastal Health, 855 West 12th Avenue, Vancouver, BC V5Z 1M9 Canada

**Keywords:** Amyotrophic lateral sclerosis, Frontotemporal dementia, Frontotemporal lobar degeneration, T-cell restricted intracellular antigen-1, TDP-43

## Abstract

Mutations in the stress granule protein T-cell restricted intracellular antigen 1 (TIA1) were recently shown to cause amyotrophic lateral sclerosis (ALS) with or without frontotemporal dementia (FTD). Here, we provide detailed clinical and neuropathological descriptions of nine cases with *TIA1* mutations, together with comparisons to sporadic ALS (sALS) and ALS due to repeat expansions in *C9orf72* (*C9orf72*+). All nine patients with confirmed mutations in *TIA1* were female. The clinical phenotype was heterogeneous with a range in the age at onset from late twenties to the eighth decade (mean = 60 years) and disease duration from one to 6 years (mean = 3 years). Initial presentation was either focal weakness or language impairment. All affected individuals received a final diagnosis of ALS with or without FTD. No psychosis or parkinsonism was described. Neuropathological examination on five patients found typical features of ALS and frontotemporal lobar degeneration (FTLD-TDP, type B) with anatomically widespread TDP-43 proteinopathy. In contrast to *C9orf72*+ cases, caudate atrophy and hippocampal sclerosis were not prominent. Detailed evaluation of the pyramidal motor system found a similar degree of neurodegeneration and TDP-43 pathology as in sALS and *C9orf72*+ cases; however, cases with *TIA1* mutations had increased numbers of lower motor neurons containing round eosinophilic and Lewy body-like inclusions on HE stain and round compact cytoplasmic inclusions with TDP-43 immunohistochemistry. Immunohistochemistry and immunofluorescence failed to demonstrate any labeling of inclusions with antibodies against TIA1. In summary, our *TIA1* mutation carriers developed ALS with or without FTD, with a wide range in age at onset, but without other neurological or psychiatric features. The neuropathology was characterized by widespread TDP-43 pathology, but a more restricted pattern of neurodegeneration than *C9orf72*+ cases. Increased numbers of round eosinophilic and Lewy-body like inclusions in lower motor neurons may be a distinctive feature of ALS caused by *TIA1* mutations.

## Introduction

We recently reported the identification of mutations in the T-cell restricted intracellular antigen-1 gene (*TIA1*) as a cause of amyotrophic lateral sclerosis (ALS) and frontotemporal dementia (FTD) [19]. Similar to several other ALS/FTD related proteins (e.g. transactive response DNA-binding protein 43 (TDP-43), fused in sarcoma (FUS) and heterogeneous nuclear ribonucleoprotein A1 (hnRNPA1)), TIA1 is an RNA binding protein that contains a C-terminal, prion-like, low complexity domain (LCD) which promotes its self-assembly and the formation of membrane-less organelles through the process of liquid-liquid phase separation (LLPS) [16, 22, 31]. Specifically, TIA1 plays a central role in the formation of stress granules (SG) that form in response to environmental stress to temporarily store and protect mRNA [1, 9, 14, 25]. SG dysfunction has been implicated in the pathogenesis of a number of neurodegenerative conditions including ALS [1, 30] and *TIA1* was previously identified as a candidate ALS gene in a yeast functional screen [5]. Moreover, a founder mutation affecting the TIA1 LCD (E384K) has been reported in Swedish/Finnish patients to cause Welander distal myopathy (WDM) [10, 15], a type of vacuolar myopathy with clinical and histopathological similarity to the myopathies caused by mutations a number of other genes that can also cause ALS/FTD (e.g. valosin containing protein and sequestosome-1) [8, 12].

In the previous study, we identified a different heterozygous missense *TIA1* mutation (P362L) in affected members of a family with autosomal dominant ALS and FTD [19]. This variant affects a highly conserved residue in the LCD and is predicted to be deleterious. Subsequent analysis of a large cohort of patients with ALS, with and without FTD, identified *TIA1* mutations in approximately 2% of familial ALS (fALS), and 0.4% of sporadic ALS (sALS), but not in neurologically normal controls [19]. Autopsy material from five *TIA1* mutation carriers showed widespread TDP-43 immunoreactive (TDP-ir) pathology as a consistent feature. Biophysical and cell culture studies demonstrated that the disease associated mutations altered phase transition of TIA1 and resulted in SG that failed to normally disassemble following the removal of stress. It is known that TDP-43 is recruited into SG under a variety of stress conditions [1] and we showed that prolonged localization of TDP-43 within persistent SG promotes TDP-43 aggregation and reduces its solubility. Based on these findings, we proposed that *TIA1* mutations are a cause of ALS and FTD; thus, reinforcing the central role of RNA metabolism and SG dynamics in the pathogenesis of this spectrum of disease [19].

Whereas the original study focused on the genetic analysis and functional effects of *TIA1* mutations, in this report we provide a more detailed description of the associated clinical features and neuropathology. In particular, we highlight phenotypic and pathological characteristics that distinguish cases with *TIA1* mutation from other types of familial and sporadic ALS and FTD.

## Materials and methods

### Case identification

**Fig. 1:**
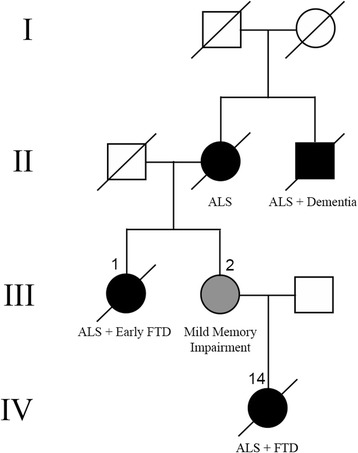
Pedigree of family UBCU2. Family of European ancestry showing an autosomal dominant pattern of inheritance of ALS ± dementia. Black symbols represent clinically affected individuals and diagonal lines indicate those who are deceased. Genetic analysis was performed on the proband (**1**), her affected niece (**14**) and her early affected sister (**2**); all of whom carried the P362L mutation in *TIA1*

Details of the genetic analysis are provided in the original report [19]. Briefly, whole exome sequencing was performed on two affected second-degree relatives who were members of a family with autosomal dominant ALS and FTD, negative for mutations in known ALS- and FTD-causing genes (UBCU2, Fig. 1). Variants that were present in a heterozygous state in both patients were filtered based on standard criteria of frequency, brain expression and predicted functional effect. The P362L missense variant in *TIA1* was determined to be the most likely candidate causal mutation, based on the protein’s normal function and structure and its association with another neurological disorder (WDM) (see above). Sanger sequencing confirmed the P362L mutation in the two affected family members and in a clinically asymptomatic family member who was an obligate carrier (UBCU2-2) (Fig. 1, Table 1). We then analyzed the *TIA1* LCD (encoded by exons 11-13) in a cohort of 1039 ALS (± FTD) patients and identified five additional *TIA1* mutations in six unrelated patients; whereas, none was identified in 3036 neurologically normal controls (*p* = 8.7 × 10^−6^). In total, nine *TIA1* mutation carriers were identified (three members of UBCU2 and six unrelated patients), representing 2.2% of fALS and 0.4% of sALS cases in our study population.

**Table 1:** Clinical and demographic characteristics of *TIA1* mutation carriers

Case	Sex	Onset (yrs)	Death or [Current Age] (yrs)	Presenting Symptom	Park	Psych	Final/Current Clinical Diagnoses	Family History	*TIA1* Mutation [19]	Post Mortem Exam
^a^UBCU2-1	F	51	55	limb weakness	no	no	ALS, early bvFTD	ALS, FTD, dementia	p.P362L	yes
^a^UBCU2-2	F	55	[55]	abnormal memory tests	no	no	CSND	ALS, FTD, dementia	p.P362L	n/a
^a^UBCU2-14	F	28	30	aphasia, behavioral changes	no	no	FTD (PPA), ALS	ALS, FTD, dementia, dyslexia	p.P362L	yes
NWU-1	F	65	68	aphasia	no	no	FTD (PPA), ALS	dementia	p.M334I	yes
TOR-1	F	73	79	aphasia	no	no	FTD (PNFA), ALS	cardiovascular disease	p.A381T	yes
ALS701-1	F	74	76	bulbar weakness	no	no	ALS, aphasia	none	p.G355R	no
ALS458-1	F	63	[64]	bulbar weakness	no	no	ALS	ALS, Parkinson’s	p.V294 M	n/a
ALS752-1	F	58	59	bulbar weakness	no	no	ALS	none	p.V360 M	yes
PITT-87	F	64	66	limb weakness	no	no	ALS	none	p.A381T	no

*ALS* amyotrophic lateral sclerosis, *bvFTD* behavioral variant FTD, *CSND* clinically symptomatic not demented, *F* female, *FTD* frontotemporal dementia, *n/a* not applicable, *Park* parkinsonism, *PNFA* progressive non-fluent aphasia, *PPA* primary progressive aphasia, not otherwise specified, *Psych* psychosis, *yrs.* years

^a^denotes members of the same family

### Clinical evaluation

Clinical information was obtained through retrospective review of the patients’ clinical records. All subjects had been evaluated by neurologists with expertise in neuromuscular disorders, behavioral neurology and/or language disorders. Clinical diagnosis of ALS was based on El Escorial criteria [3]. All patients signed informed consent and this study was approved by the ethics committee of all respective institutions.

### Neuropathology

Post mortem examination restricted to the central nervous system had been performed on five of the affected *TIA1* mutation carriers (Tables 1 and 2). In four of the five cases, the entire spinal cord was available for examination; whereas, in case NWU-1, only the upper segments of cervical spinal cord were available. Microscopic evaluation was performed on 5 μm-thick sections of formalin fixed, paraffin-embedded material representing a wide range of anatomical regions (Table 2). Histochemical stains included hematoxylin and eosin (HE), HE combined with Luxol fast blue (HE/LFB), modified Bielschowsky silver stain, Gallyas silver stain, Masson trichrome, Periodic Acid Schiff with and without diastase, Alcian Blue (pH 2.5) and Congo red. Standard immunohistochemistry (IHC) was performed using the Ventana BenchMark XT automated staining system with primary antibodies against alpha-synuclein (Thermo Scientific; 1:10,000 following microwave antigen retrieval), beta amyloid (DAKO; 1:100 with initial incubation for 3 h at room temperature), hyperphosphorylated tau (clone AT-8; Innogenetics, Ghent, Belgium; 1:2000 following microwave antigen retrieval), phosphorylation-independent TDP-43 (ProteinTech; 1:1000 following microwave antigen retrieval), ubiquitin (DAKO; 1:500 following microwave antigen retrieval), FUS (Sigma-Aldrich; 1:1000 following microwave antigen retrieval), p62 (BD Biosciences; 1:500 following microwave antigen retrieval), poly-(A) binding protein (PABP; Santa Cruz; 1:200 following microwave antigen retrieval), hnRNP A1 (Santa Cruz; 1:100 following microwave antigen retrieval), hnRNP A3 (Sigma-Aldrich; 1:100 following microwave antigen retrieval) and hnRNP A2/B1 (Santa Cruz; 1:500 following microwave antigen retrieval). In addition, we tested a number of commercial monoclonal and polyclonal antibodies against different epitopes of human TIA1, raised in different species (Table 3).

**Table 2:** Semiquantitative analysis of neurodegeneration and TDP-ir pathology in *TIA1* mutation carriers

		Neurodegeneration					TDP-43 Immunohistochemistry				
		NWU-1	TOR-1	UBCU2-14	UBCU2-1	ALS752-1	NWU-1	TOR-1	UBCU2-14	UBCU2-1	ALS752-1
		FTD, ALS	FTD, prob. ALS	FTD, prob. ALS	ALS, early FTD	ALS	FTD, ALS	FTD, prob. ALS	FTD, prob. ALS	ALS, early FTD	ALS
pyramidal motor system	motor cortex	+	+	+	++	+	+	++	+	+	+++
	CN XII	+++	++	+++	++	+++	++	+++	++	++	++
	CST	++	+	+++	++	++	n/a	n/a	n/a	n/a	n/a
	ant. horn	++	++	+++	+++	+++	++	++	+++	+++	+++
neocortex	prefrontal	++	+	+++	++	–	+++	+++	+++	+++	++
	temporal	+	+	++	+	–	++	++	++	++	++
	parietal	–	–	++	+	–	+++	++	++	++	+
striatonigral system	caudate	+	–	++	+	–	+	++	+++	+++	+++
	putamen	–	–	–	–	–	+	+	++	+++	+
	GP	–	–	–	–	–	+	+	+	+	+
	SN	+++	+	+++	++	++	++	++	+++	+++	++
limbic system	hip. CA	–	–	–	–	–	+	+	+	+	+
	hip. dentate	++	+	–	–	–	++	+++	++	++	++
thalamus		+	–	–	–	–	+	–	+	–	+
midbrain	PAG	+	–	++	+	–	+	+	++	+	+
cerebello-pontine system	cb cortex	–	–	–	–	–	–	–	–	–	–
	cb dentate	+	–	–	–	–	–	–	–	–	–
	basis pontis	–	–	–	–	–	+	–	+	–	+

The patients are ordered according to their earliest and/or predominant clinical features from predominant FTD (left) to pure ALS (right). *ALS* amyotrophic lateral sclerosis, *ant*. anterior, *cb* cerebellar, *CA* cornu ammonis, *CN XII* twelfth cranial nerve (hypoglossal) nucleus, *CST* corticospinal tract, *deg*. non-specific changes of chronic degeneration, *FTD* frontotemporal dementia, *GP* globus pallidus, *hip*. hippocampal, *n/a* not applicable, *PAG* periaqueductal grey matter, *prob*. probable, *SN* substantia nigra. Semiquantitative grading of pathology; −, none; +, mild; ++, moderate; +++, severe

**Table 3:** TIA1 antibodies used for immunohistochemistry and double label immunofluorescence

Antibody	Species	Epitope	Dilution
Santa Cruz #sc-1751	Goat polyclonal (clone C-20)	near C-terminus	1:300
Santa Cruz #sc-48371	Mouse monoclonal (clone D-9)	aa 21-140 (near N-terminus)	1:200
Santa Cruz #sc-28237	Rabbit polyclonal (clone H-120)	aa 21-140 (near N-terminus)	1:300
Abcam #ab140595	Rabbit monoclonal	aa 350 to the C-terminus	1:100
Abcam #ab40693	Rabbit polyclonal	aa 350 to the C-terminus	1:500
ProteinTech #12133-2-AP	Rabbit polyclonal	human TIA1-GST fusion protein	1:100

#, catalogue number; *GST* glutathione S-transferase

Double-labelling immunofluorescence (IF) experiments were performed on paraffin-embedded tissue sections that were heated to 60°C for 20 min, then immediately deparaffinized and rehydrated. Antigen retrieval was performed in citrate buffer (10 mM, pH 6.0, 10 min at 95°C in a water bath). The sections were blocked for 1 h with 5% donkey serum in 0.1% triton X-100 in TBS. Incubation with various combinations of primary antibodies was performed in the same blocking solution overnight at 4C. The combinations included a rat anti-phosphorylated TDP-43 (from M. Neumann, 1:1000) [24] with one of three anti-TIA1 antibodies: Santa Cruz goat anti-TIA1 (1:300), Santa Cruz rabbit anti-TIA1 (1:300) or Proteintech rabbit anti-TIA1 (1:100) (Table 3).The sections were then washed, and incubated with appropriate Alexa Fluor- or biotin-conjugated secondary antibodies at 1:1000 dilution for 1 h at room temperature. When needed, a third step with Alexa Fluor-conjugated streptavidin (1:1000) was added for 40 min. Background fluorescence was then quenched by staining with 0.1% Sudan Black in 70% ethanol for 15 min. Slides were mounted after a 15-min incubation in DAPI with Prolong-Gold anti-fade reagent (Invitrogen). Microscopy was performed using a Nikon Eclipse i-80 epifluorescent microscope and NIS-Elements software. Images were further processed and merged using Image J.

The severity of chronic degenerative changes and the burden of TDP-ir pathology in different brain regions was evaluated using a semi-quantitative scoring system, as follows: −, absent; +, mild/few (easy to find but not present in every medium power field); ++ moderate (at least a few in most fields); +++, severe/numerous (many in virtually every field). In addition, the number of lower motor neurons (LMN) (defined as medium to large cells with prominent peripheral Nissl substance) and the number of LMN containing various types of neuronal cytoplasmic inclusions (NCI), including Bunina bodies, round inclusions and cored Lewy body-like inclusions (LBLI) seen with HE stain, as well as TDP-ir NCI with granular, filamentous or compact morphology were counted in sections of cervical and lumbosacral spinal cord in the cases with *TIA1* mutations (*N* = 5) and in sections of sALS and *C9orf72*+ ALS (*N* = 10 each). In each case, one slide from each of the available tissue blocks representing different levels of cervical or lumbar spinal cord enlargement was evaluated and the counts averaged (mean number per tissue section). Similar evaluation was performed on sections of medulla; however, this data was not included in the statistical analysis because the variation in the anatomical level resulted in significant differences in the representation of the hypoglossal nucleus among cases.

### Statistical analysis

Statistical analysis and data graphing were performed using GraphPad Prism 6.0 software. Non-parametric Kruskal-Wallis test with Dunn’s multiple comparison test were used to analyze the differences among the groups in round eosinophilic and cored LBLI; whereas, ANOVA with multiple comparisons was used to analyze the differences in TDP-ir NCI in LMN.

## Results

### Clinical features

Brief summaries of the demographic and clinical information are provided in Table 1. Below are detailed descriptions of each case.

#### UBCU2-1

This previously healthy woman came from a family of European ancestry (Fig. 1) [19]. Her mother had ALS onset at age 51 and died at age 56 and her maternal uncle died in his 60s of ALS with dementia. At age 51, UBCU2-1 developed progressive, asymmetric upper limb weakness and occasional tripping. Initial examination found mild-to-moderate atrophy and weakness of upper and lower limb muscles, without fasciculations. Deep tendon reflexes were symmetrically brisk. Bulbar musculature was intact. Nerve conduction studies of median and ulnar nerves showed low amplitude but normal conduction velocities; whereas, sensory studies were normal. EMG showed denervation of several muscles in all four extremities and of paraspinal muscles. She was felt to be cognitively normal at that time but was not formally tested. A clinical diagnosis of definite ALS was made.

Over the subsequent 4 years, her extremity weakness progressed to the point of requiring full assistance for daily activities. She became increasingly dysarthric and had difficulty breathing, but was able to swallow. A change in personality was first noted 6 months before her death when she became disinhibited, mildly disoriented, inappropriately emotional and repetitive. She died at age 55 with a clinical diagnosis of ALS and early behavioral variant FTD (bvFTD). An autopsy limited to brain and spinal cord was performed. Neuropathological examination showed ALS-TDP and FTLD-TDP (subtype B) and tau-ir neurofibrillary tangles restricted to the entorhinal and transentorhinal cortex (Braak stage I).

#### UBCU2-2

The sister of the proband was assessed at age 55. Neither she nor her family reported any motor or cognitive symptoms. Clinical neurological assessment was normal; however, detailed neuropsychological assessment found deficits on measures of verbal and non-verbal memory. She was considered symptomatic (early memory abnormalities), but not demented.

#### UBCU2-14

The niece of the proband developed difficulty with word-finding at age 28. Around the same time, her family noted that she was becoming emotionally flat, withdrawn, apathetic and displaying little empathy. A few months later she developed dysarthria, difficulty chewing and swallowing and she became clumsy and prone to minor injury. Language comprehension began to decline, she was increasingly forgetful and had difficulty planning and organizing simple household tasks. She had suffered from dyslexia since childhood, as had several of her paternal relatives. When initially evaluated, 6–9 months following her disease onset, her Mini-Mental Status Examination (MMSE) score was 21/30, the Montreal Cognitive Assessment test (MoCA) showed prominent visuospatial dysfunction and the Frontal Assessment Battery (FAB) was 3/15. There was mild emotional incontinence. She had a slight spastic dysphonia and mild Gegenhalten in the right arm and leg. MRI demonstrated moderate symmetric frontotemporal atrophy.

When next evaluated, at age 29, she was found to have more severe cognitive impairment (MMSE score of 11/30 and MoCA score of 8/30). She was friendly but apathetic and emotionally blunt. Her speech was anomic, dysarthric and perseverative, but grammatically intact. She could follow simple commands and repeat sentences. Although her aphasia was difficult to classify, it was felt to have features of apraxia of speech and progressive non-fluent aphasia (PNFA). Physical examination was limited by her inability to fully cooperate. Strength of her facial muscles was normal, but tongue movement was impaired. Jaw jerk was brisk and she was hyperreflexic and mildly spastic in all extremities. Muscle bulk and strength were normal in all extremities and no fasciculations were noted. EMG showed active denervation, fibrillation potentials, irritability and some positive sharp waves in several muscles of both legs; however, due to patient noncompliance, the EMG study was discontinued before the upper extremities could be tested. The clinical diagnosis was that of primary progressive aphasia (PPA, difficult to classify) and “probable” ALS (due to the incomplete EMG study).

She progressed rapidly and became almost mute with a very limited degree of language comprehension within the next 6 months. Her bulbar dysfunction worsened and the right lower extremity became weak. She died at age 30 with a clinical diagnosis of FTD with both behavioral symptoms and progressive aphasia and probable ALS. Autopsy limited to brain and spinal cord demonstrated ALS-TDP and FTLD-TDP (subtype B).

#### NWU-1

This woman presented at age 65 with intermittent confusion and aphasia characterized by laconic speech, word finding difficulties and paraphasic errors in writing, but with intact language comprehension. No motor features were identified at that time and she was given a preliminary clinical diagnosis of primary progressive aphasia (PPA).

More detailed evaluation at age 67 found apraxia of speech, dysarthria, telegraphic phrases, anomia, problems with sentence comprehension and agrammatic writing. There were also impairments in executive function, motivation and insight. Motor examination demonstrated bulbar weakness, but normal limb strength and reflexes without fasciculations. MRI showed extensive cerebral white matter hyperintensities, attributed to chronic ischemia, and SPECT scan showed mild hypoperfusion of the left anterior temporal lobe.

Her disease progressed rapidly and by age 68 she had global aphasia, swallowing difficulties and fasciculations in the tongue and all limbs. EMG revealed findings of motor neuropathy and spontaneous motor activity, and swallowing studies were abnormal. She died later that year. Her family history was positive for late onset dementia, but not for ALS.

An autopsy was performed but was limited to the brain. Neuropathological examination showed FTLD-TDP (type B) and ALS-TDP pathology in the brainstem and high cervical spinal cord. There was very mild Alzheimer-type pathology with rare neuritic senile plaques and neurofibrillary tangles (Braak stage II).

#### TOR-1

This woman presented at age 73 with speech abnormalities characterized by frequent errors in grammar and syntax. Her speech progressively deteriorated and she also developed swallowing difficulties with frequent choking. Neuroimaging studies were unremarkable and she was diagnosed with PNFA and probable ALS. She developed depressive symptoms, but no other behavioral abnormalities. Her family history was negative for neurological disorders. Neuropathological examination showed ALS-TDP and FTLD-TDP (type B).

#### ALS701-01

This 75-year old woman presented with approximately 7 months of bulbar weakness and pseudobulbar affect and was diagnosed with clinically definite ALS. Her ALS Functional Rating Scale (ALSFRS) score was 25/48 and the ALS Cognitive Behavioral Screen (ALS-CBS) was compatible with probable frontotemporal cognitive impairment with expressive aphasia. She died at age 76. There was no family history of ALS, dementia or Parkinson disease. Post-mortem examination was not performed.

#### ALS458-01

This woman presented at age 63 with progressive bulbar weakness. By age 64 she met clinical criteria for definite ALS and required ventilatory support. Her ALSFRS score was 29/48. ALS-CBS was normal and she did not have any cognitive or behavioral symptoms. She was subsequently lost to follow up. Family history was positive for ALS and Parkinson disease.

#### ALS752-01

This woman presented at age 58 with 4 months of bulbar symptoms and was diagnosed with clinically definite ALS. Her ALSFRS score was 43/48 and ALS-CBS scores were normal. She died 21 months after disease onset. There was no family history of ALS, dementia or Parkinson disease. Autopsy limited to brain and spinal cord showed ALS-TDP and mild TDP-43 pathology in the extramotor cerebral cortex.

#### PITT-87

This woman presented at age 64 with sudden onset of bilateral leg weakness and back pain. EMG performed 4 months later showed widespread denervation in the legs and thoracic paraspinal muscles; however, weakness was only demonstrated in the distal legs. Over the following year, her weakness became more severe and spread to involve proximal legs, arms and face with a hyperactive jaw jerk and increased tone in the legs. Her respiratory function declined to a forced vital capacity of 33%. Cognitive testing performed at age 65 was normal with MMSE 30/30 and ALSCBS 19/20. She died at age 66. There was no history of neurological disorders in the family. Post-mortem examination was not performed.

### Neuropathology

#### Gross pathology

The fresh brain weight ranged from 1132 to 1450 g (mean 1300 g) with two cases showing bifrontal lobar atrophy (UBCU2-1 and UBCU2-14) and one with left side predominant frontotemporal atrophy (TOR-1). The hippocampi were normal in size and only one case showed mild atrophy of the head of the caudate nucleus. Mild or moderate reduction in the pigmentation of the substantia nigra was noted in four cases.

#### General histology

**Fig. 2:**
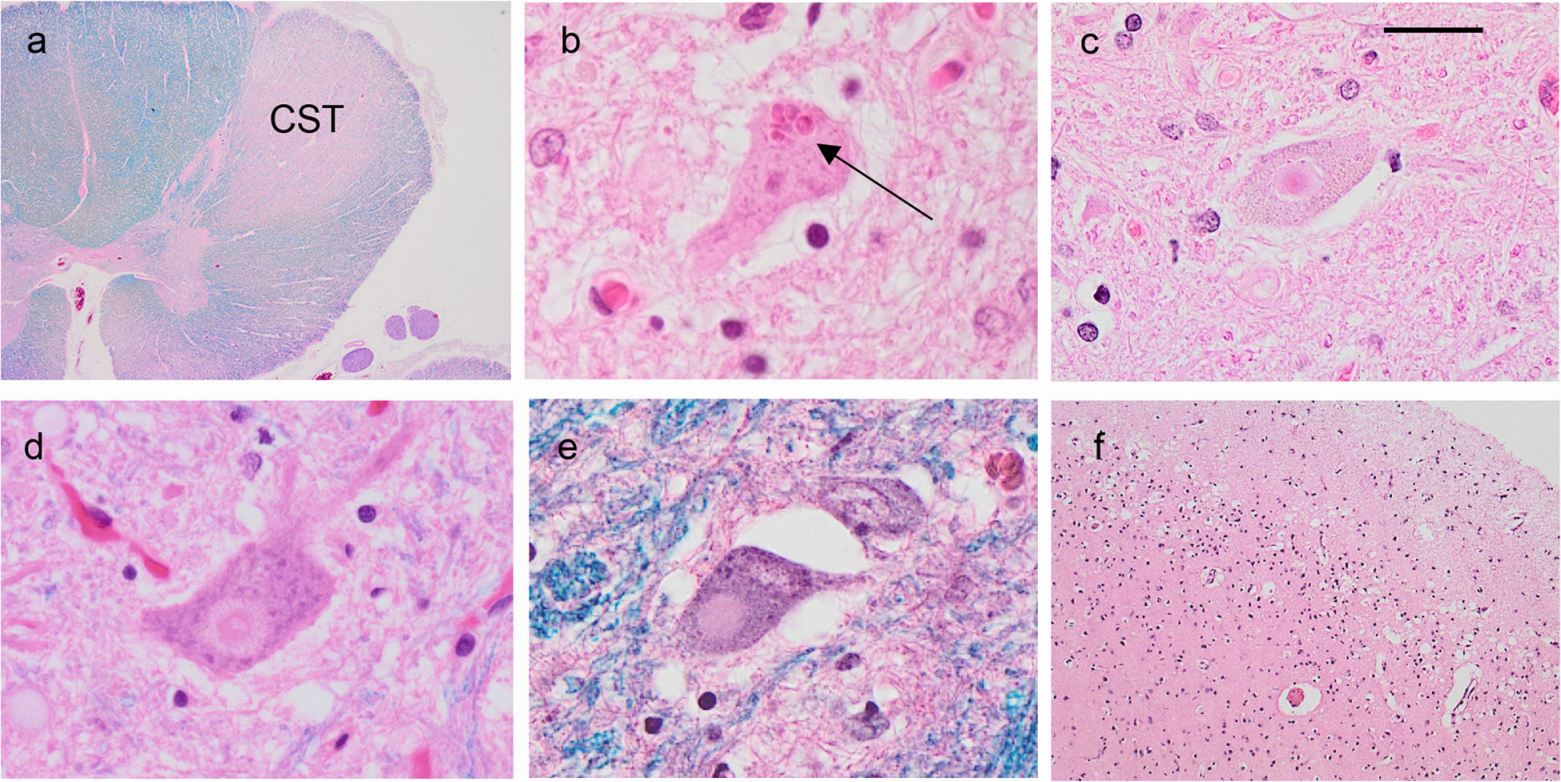
Histological changes in *TIA1* mutation carriers. Cross section of spinal cord showing severe loss of myelin stain in the corticospinal tracts (CST) (**a**). Lower motor neurons containing Bunina bodies (arrow) (**b**) and large, round cored Lewy body-like inclusions (LBLI) (**c**, **d**) or round eosinophilic inclusions without distinct cores (**e**) were present in the medulla and spinal cord. Extra-motor pathology included chronic degeneration with superficial, laminar microvacuolation of the prefrontal cortex (**f**). **a** and **e**, HE/LFB stain; **b** - **d** and **f**, HE stain. Scale bar: **a**, 1200 μm; **b**, 7 μm, **c**, 23 μm; **d** and **e**, 15 μm; **f**, 205 μm

The pyramidal motor system showed chronic degenerative changes in all cases (Table 2). The primary motor cortex tended to show mild neuronal loss and reactive changes, there was variable axonal and myelin loss in the corticospinal tracts (Fig. 2a) and moderate or severe loss of LMN in the brainstem and spinal cord. In all cases, small, brightly eosinophilic Bunina bodies were present in some of the remaining LMN (Fig. 2b). In addition, all cases were found to have some LMN containing sharply demarcated, round cytoplasmic inclusions that were often larger than the cell nucleus (Fig. 2c-e). These were pale pink or amphophilic with HE stain and approximately half had a compact central core, surrounded by a paler halo, similar in appearance to a Lewy body (Lewy body-like inclusion, LBLI). These round inclusions were distinct from the more irregularly shaped and more brightly eosinophilic hyaline inclusions that are frequently found in LMN in both ALS and normal aging (not shown). They were also easily identified on HE/LFB stained sections but did not stain with other histochemical stains such as Masson trichrome, Periodic Acid Schiff, Congo red or silver stains. Round inclusions were present in LMN of both the spinal cord ventral grey matter and the hypoglossal nucleus but were not seen in other neuronal populations. They averaged approximately two per tissue section, with a maximum frequency of 5 in one section (see quantitation below).

The four cases with clinical features of FTD also showed degeneration of the extramotor neocortex, with the prefrontal regions being most consistently and severely affected (Table 2, Fig. 2f). Apart from the pyramidal system motor nuclei, the only other subcortical regions that commonly showed degeneration were the caudate nucleus, periaqueductal grey matter and substantia nigra. The hippocampus was usually spared and no case showed selective loss of CA1 pyramidal neurons (hippocampal sclerosis).

#### TDP-43 immunoreactive pathology

**Fig. 3:**
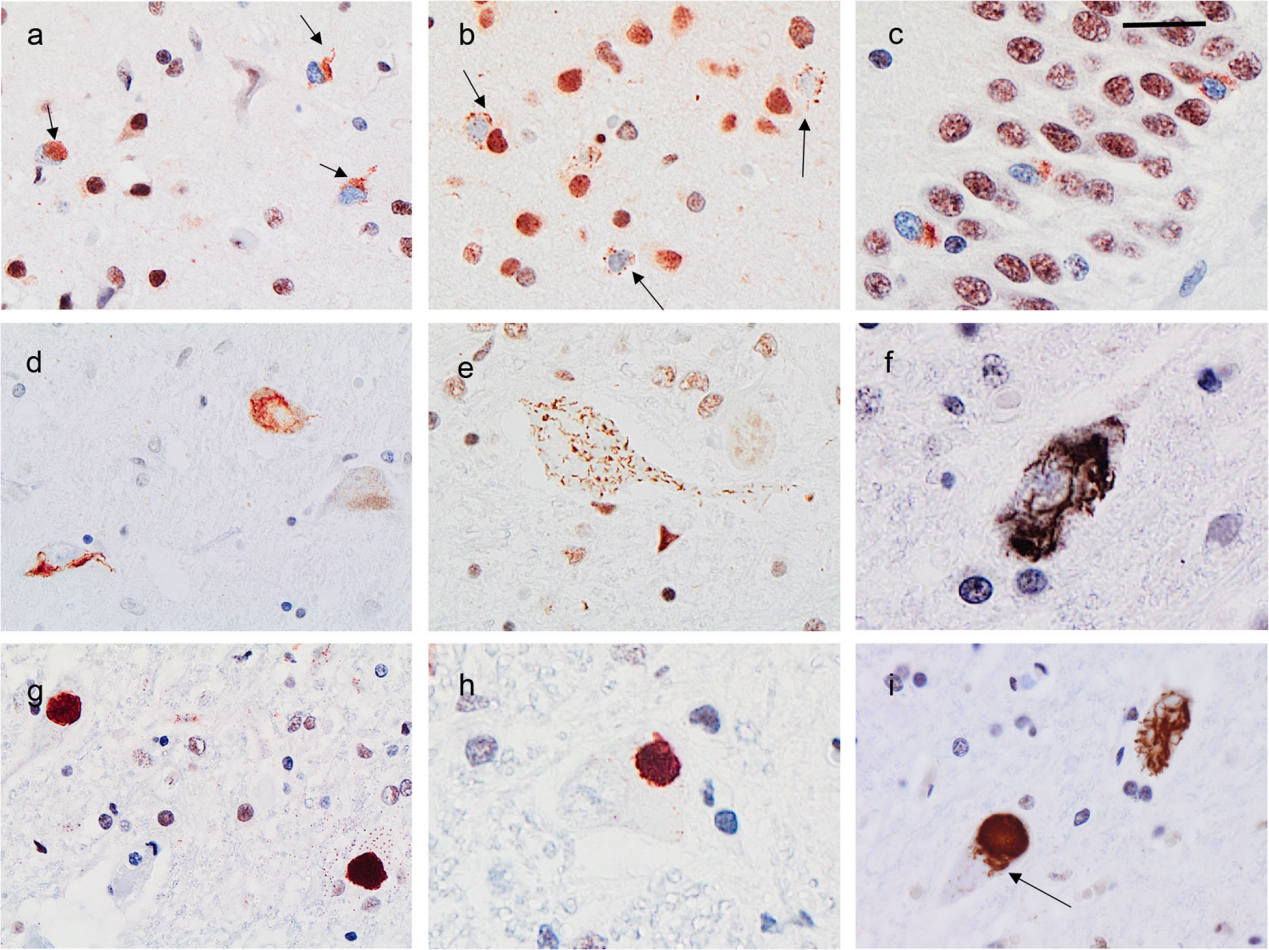
TDP-43 immunoreactive pathology in *TIA1* mutation carriers. Numerous predominantly granular TDP-43 immunoreactive (TDP-ir) neuronal cytoplasmic inclusions (NCI, arrows) were present in the prefrontal cortex (**a**) and primary motor cortex (**b**). Hippocampal dentate granule cells (**c**) and dopaminergic neurons in the substantia nigra (**d**) were consistently affected in all cases. Lower motor neurons (LMN) of the medulla and spinal cord (**e** – **i**) contained NCI that were granular (**e**), filamentous (**f**) or round and compact (**g** and **h**). Single LMN containing combinations of NCI types were not uncommon (**i**, arrow points to filamentous inclusions in close proximity to a compact, round NCI). Phosphorylation-independent TDP-43 immunohistochemistry. Scale bar: **a** and **i**, 25 μm; **b**, **c** and **e**, 18 μm; **d** and **g**, 36 μm; **f** and **h**, 9 μm

TDP-ir pathology tended to be more severe and anatomically widespread than the degenerative changes (Table 2). There was moderate to severe involvement of the extramotor cerebral neocortex with the prefrontal cortex being most severely affected. In all cases, the pattern of pathology was most consistent with FTLD-TDP subtype B [18] with NCI in all cortical layers that were more often granular than compact (Fig. 3a). There were relatively few short thick dystrophic neurites (DN), but wispy threads and dot like structures were often concentrated in layer II. There were no neuronal intranuclear inclusions. Similar TDP-ir pathology was present in the primary motor cortex (Fig. 3b), but was milder in all cases with the exception of the patient with clinically pure ALS (ALS752-1) (Table 2). The hippocampus showed moderate numbers of TDP-ir NCI in the dentate granule cells (Fig. 3c) but few in the pyramidal layer and no TDP-ir wispy threads in the CA1/subiculum, characteristic of hippocampal sclerosis. Varying degrees of TDP-ir granular NCI and mild DN pathology was a consistent finding throughout the basal ganglia and substantia nigra and periaqueductal grey matter (Table 3, Fig. 3d); whereas, the thalamus and pons were only mildly and inconsistently involved and the cerebellum was spared.

**Fig. 4:**
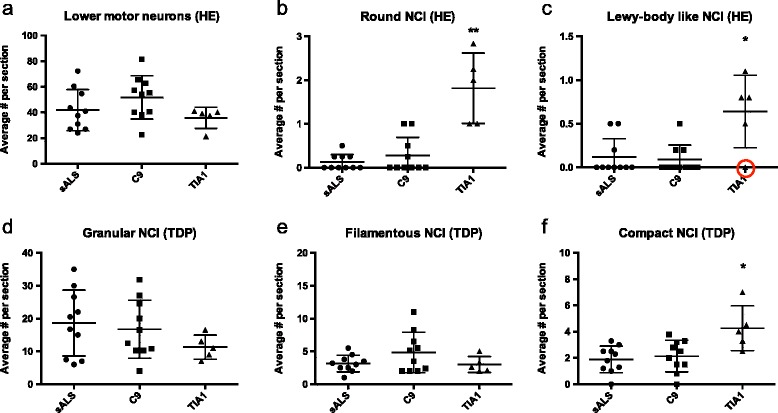
Quantitative comparison of spinal cord lower motor neurons (LMN) and different types of neuronal cytoplasmic inclusions (NCI) in LMN in ALS cases with *TIA1* mutations, the *C9orf72* mutation (C9) and sporadic ALS (sALS). Quantitation in **a**-**c** was performed on HE stained sections; whereas **d**–**f** was based on TDP-43 immunohistochemistry. There was no difference in the number of spinal cord LMN among groups (**a**). Round NCI (**b**) and Lewy body like inclusions (**c**) were significantly more frequent in *TIA1* mutation carriers than in either C9 or sALS cases. In **c**, the red circle denotes the data point corresponding to the case with only a single section of upper cervical cord available for evaluation. No differences were seen among groups in the number of granular (**d**) or filamentous (**e**) TDP-43 NCI; however, round compact NCI were more frequent in *TIA1* mutation carriers (**f**). (** = *p* < 0.005, * = *p* < 0.05 compared to C9 and sALS groups, respectively)

Many of the remaining LMN in the spinal cord and hypoglossal nucleus contained TDP-ir NCI of varying morphology (Fig. 3e-i). Most common were small granules, diffusely distributed throughout the perikaryal cytoplasm (Figs. 3e, and 4). Filamentous NCI were also present (Fig. 3f), but were less common than large compact NCI that were often round and of similar size to the round/LBL inclusions seen on HE (Figs. 3g-i and 4). It was not uncommon to find a LMN that contained more than one inclusion type (Fig. 3i).

TDP-ir glial inclusions were not quantified separately but were a common feature in affected grey matter regions as well as the subcortical white matter. They were relatively uncommon in the corticospinal tracts and rare in other spinal cord funiculi.

#### Other pathological findings

Most cases did not show any other significant neurodegenerative pathology. Mild Alzheimer-type pathology was present in two cases with tau-ir neurofibrillary tangles limited to the entorhinal and transentorhinal cortex (Braak stage I or II, UBCU2-1 and NWU-1) and infrequent neuritic senile plaques (CERAD rare, NWU-1). No alpha-synuclein-ir Lewy bodies or neurites were present.

#### Comparison of pyramidal motor system pathology in ALS patients with and without TIA1 mutations

No differences were seen in the degree of chronic neurodegeneration in the primary motor cortex, corticospinal tracts, hypoglossal nucleus or ventral grey matter of the cervical or lumbar spinal cord when comparing ALS patients with *TIA1* mutations, *C9orf72* mutations and sALS without either mutation (LMN counts shown in Fig. 4a). There were also no differences in the burden of TDP-ir pathology in the primary motor cortex or the numbers of LMN containing Bunina bodies (data not shown). However, cases with *TIA1* mutations had significantly more round inclusions and LBLI seen on HE stained sections of spinal cord than either the sALS or *C9orf72*+ group (Fig. 4b and c, *p* < 0.005 and *p* < 0.05, respectively). This difference was all the more striking given that the analysis included the *TIA1* mutation case with only a single section of high cervical spinal cord available for examination (NWU-1). Although that case had only one round inclusion and no LBLI in the single cervical section (Fig. 4c, red circle), multiple round and LBLI were present in the hypoglossal nucleus. On average, one to two round inclusions were present in each section of spinal cord and medulla from the *TIA1* mutation carriers; whereas, in the non-*TIA1* cases, most sections did not have any round inclusions and cored LBLI were exceptionally rare.

With TDP-43 IHC, granular LMN NCI were the most frequent type in all three patient groups, representing 60-75% of the total. No differences were found among the groups in the frequency of granular or filamentous NCI (Fig. 4d and e); however, the *TIA1* mutation cases had significantly more compact NCI (*p* < 0.05) than *C9orf72*+ or sALS cases (Fig. 4f).

#### Immunostaining for TIA1, other SG components and other RNA-binding proteins (RBP)

**Fig. 5:**
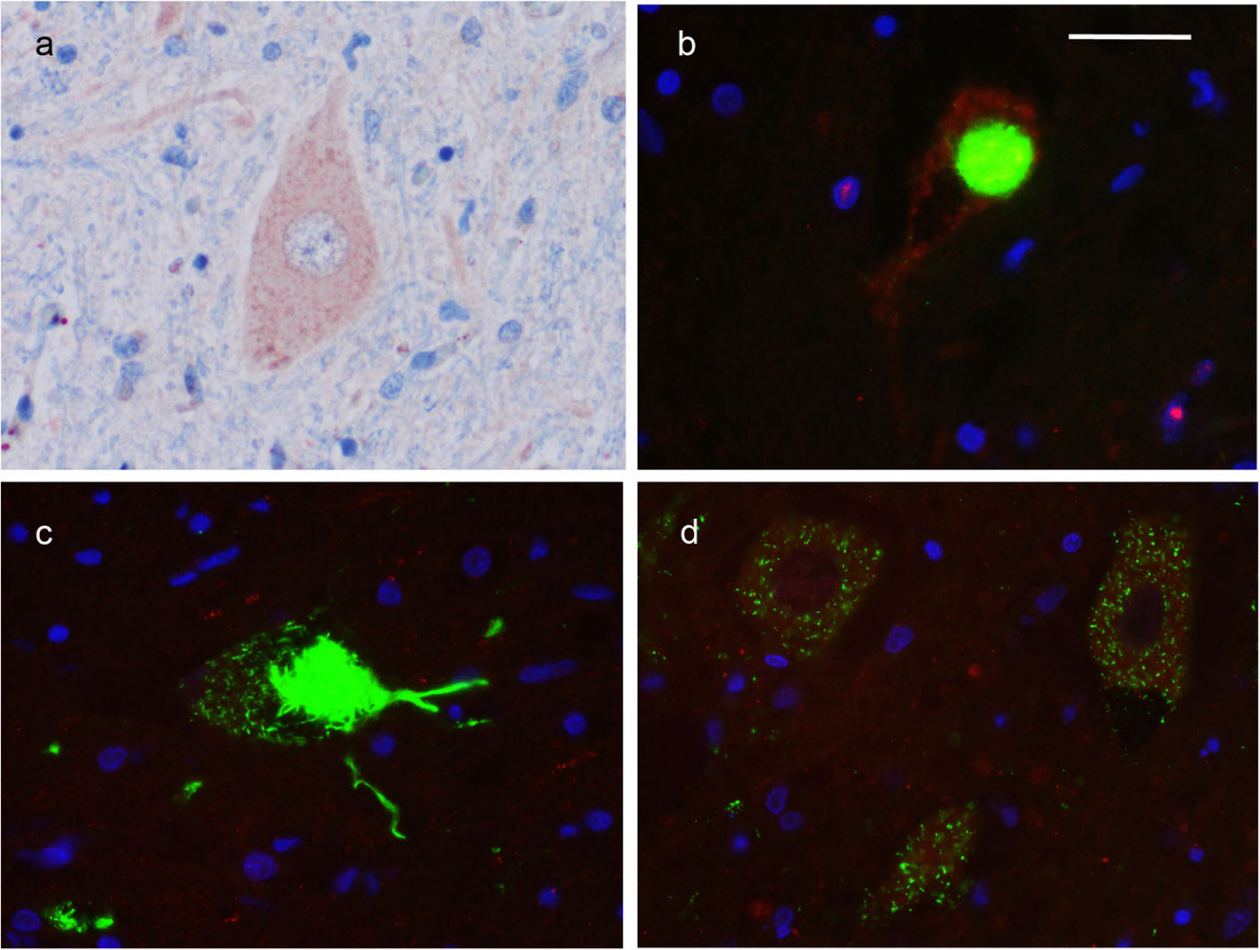
TIA1 immunohistochemistry (IHC) and double-label immunofluorescence (IF) of lower motor neurons from cases with *TIA1* mutations. Representative image of TIA1 IHC with the rabbit polyclonal (Santa Cruz #sc-28,237, clone H-120) antibody showing delicate granular cytoplasmic staining (**a**). Double label IF with pTDP (green) and TIA1 (red) antibodies failed to show co-localization of TIA1 in compact (**b**), filamentous (**c**) or granular pTDP-43-ir NCI (**d**). Scale bar: **a** and **d**, 24 μm; **b** and **c**, 16 μm

IHC using a number of anti-TIA1 primary antibodies that recognize different TIA1 epitopes (Table 3) failed to demonstrate any abnormality. Most of the antibodies showed moderately intense diffuse staining of neuronal cytoplasm and some also stained the nucleus, although none showed preferential nuclear staining (Fig. 5a). The staining patterns were similar in ALS cases with and without *TIA1* mutations and in sections from normal controls. Specifically, no TIA1-ir pathological inclusions were demonstrated in cases with *TIA1* mutations. For double label IF an antibody that recognizes phosphorylated pathological TDP-43 (pTDP-43) was used (to avoid the normal nuclear positivity) in combination with each of the three TIA1 antibodies that gave the best results with IHC. The same types of NCI were labelled for pTDP-43 as had been seen on light microscopy with TDP-43 IHC (Fig. 5b-d). The TIA1 antibodies again showed diffuse cytoplasmic +/− nuclear reactivity; however, there was no specific co-localization of TIA1 with compact, filamentous or granular pTDP-43-ir NCI (Fig. 5b-d). IHC using antibodies against another SG marker (PABP) and against a number of other RBP (FUS, hnRNPA1, hnRNPA3 and hnRNPA2B1) also failed to show any distinctive staining pattern in the *TIA1* mutation cases (data not shown).

## Discussion

The purpose of this report is to provide a detailed description of the clinical and neuropathological features associated with the recently identified *TIA1* mutations that cause ALS ± FTD. Although the number of cases currently available is not extensive, it is sufficient to identify some features that may be helpful in distinguishing these cases from other ALS subtypes.

In the initial discovery series, *TIA1* mutations were identified in 2.2% of familial ALS and 0.4% of sporadic ALS cases [19]; far less frequent than the most common genetic cause of ALS, which is the *C9orf72* repeat expansion (responsible for 37% of familial and 6% sporadic ALS [26]), but similar to the frequencies reported for *VCP* and *SQSTM1* mutations and more common than many other genetic variants that have been associated with ALS [2]. The mean age of disease onset in our cases was 60 years, with an average disease duration of 3 years; similar to what has been reported for ALS caused by the *C9orf72* mutation and for cases of ALS with no identified mutation [28]. Only three of our seven probands (43%) had a family history of neurological disease, which is significantly less than for *C9orf72* and *SOD1* mutations [28] and suggests that *TIA1* mutations may be variably penetrant. Interestingly, all of our *TIA1* mutations carriers were female, despite there being no sex bias in the case series in which they were identified. This finding is all the more striking, given that ALS, in general, is reported to be more common in men with a ratio of males to females of 1.7:1 [21] and could indicate that some factor associated with biological sex affects the penetrance of *TIA1* mutations, similar to what has been reported for some other genetic subtypes of ALS/FTD [6]. This association will need to be investigated further in additional case series and we note that one affected member of UBCU2 was male, although no source of DNA was available to confirm his genetic status.

The initial clinical features of our *TIA1* mutation carriers varied, with five presenting with symptoms of ALS, three with early changes of FTD and one who was asymptomatic but found to have abnormal memory function of neuropsychological testing (Table 1). Of the eight cases that developed ALS symptoms, bulbar weakness was a prominent feature in six (75%), which is more common than in cases of sALS (26%) or those with the *C9orf72* mutation (44%) [28]. Of the five patients who presented with, or later developed FTD, the initial and most prominent problem was expressive aphasia in four, whereas only one patient showed mainly behavioral abnormalities. The high frequency of primary progressive aphasia (PPA) in our *TIA1* mutation carriers is different from those with the *C9orf72* mutation whose FTD phenotype is more often bvFTD [11]. It is important to note that the case series used to identify mutation carriers had a primary ALS diagnosis with only a small proportion (< 4%) also having FTD, which may underestimate the incidence of *TIA1* mutations in patients with mainly FTD. Interestingly, none of our *TIA1* mutation carriers developed significant parkinsonism or psychotic features, both of which are commonly reported in series with the *C9orf72* mutation [11, 27].

It is intriguing that another founder mutation affecting the LCD of *TIA1* (E384K) was previously identified in Swedish and Finnish populations to cause WDM, which is characterized by late-onset slowly progressive weakness of hand and distal leg muscles and is associated with a rimmed vacuolar myopathy which is TDP-ir [10, 15]. To our knowledge, no patients with WDM have been reported to also develop ALS or FTD and none of our ALS patients with other *TIA1* mutations had a personal or family history of muscle disease. It is possible that something about the specific amino acid substitution caused by the E384K mutation leads to a distinct and selective phenotype, or that the expression is influenced by other genetic factors common to the specific ethnic population in which the WDM *TIA1* mutation occurs [15]. However, given the fact that the E384K mutation is reported to show similar effects to the ALS associated *TIA1* mutations in biophysical and cell culture studies [19], it is also possible that greater overlap in the clinical features exist but has not yet been recognized due to referral or reporting biases. This might not be unexpected given the number of other genes (including *VCP*, *SQSTM1*, *HNRNPA1*, *HNRNPA2B1* and *MATR3*), in which mutations cause clinical syndromes, referred to as multisystem proteinopathies, with ALS, FTD, inclusion body myopathy and bone disease each showing variable penetrance [29].

Overall, the neuropathology of our patients with *TIA1* mutations was characterized by chronic degenerative changes, primarily involving the pyramidal motor system, prefrontal neocortex and substantia nigra, with more anatomically widespread TDP-ir pathology (Table 2, Figs. 2 and 3). In all four cases with clinical FTD, the pattern of neocortical TDP-ir pathology fit best with FTLD-TDP type B and was similar to what is found in most patients with FTD combined with ALS, including those with the *C9orf72* mutation [18]. None of the *TIA1* mutation cases showed severe degeneration of the caudate nucleus or hippocampal sclerosis which are both common in other genetic causes of FTLD-TDP (e.g. *C9orf72* and *GRN* mutations) [11, 20].

The most striking aspect of the pyramidal system pathology in the *TIA1* mutation carriers was the number of round, sometimes LBL inclusions seen in LMN of the spinal cord and medulla with HE stain (Fig. 2). Although similar round inclusions were also found in some cases of sALS and *C9orf72*+ cases, they never averaged more than one per tissue section and were completely absent in most cases. In contrast, round and LBL inclusions were a consistent feature in the *TIA1* mutation carriers, with at least one, and often multiple examples, present in each section of spinal cord and medulla. Consistent with this was the finding of significantly more compact round TDP-ir NCI in LMN in the *TIA1* mutation cases, which were often of a similar size and shape as the round/LBL inclusions seen on HE stain. This finding suggests that frequent round/LBLI in LMN may represent a pathological signature of ALS-TDP caused by *TIA1* mutations and that the formation of these particular inclusion bodies may somehow be related to the altered SG dynamics that has been shown to be associated with expression of the mutant *TIA1* protein [19]. Although this is somewhat speculative, it is interesting that cases of ALS caused by mutations in the RNA-binding protein FUS are also characterized by large round cytoplasmic inclusions in LMN that are visible with HE stain (although generally more basophilic) and that these have been proposed to form in persistent SG [7]. Round, hyaline, LBLI have also been described in some (but not all) cases of ALS caused by *SOD1* mutations [13, 23], where they are composed of misfolded SOD1, rather than TDP-43, and may be induced by ER stress [33].

The pathomechanism that has been proposed for ALS associated with *TIA1* mutations is that the amino acid change in the LCD enhances its intermolecular interaction, which promotes LLPS and results in SG that are more persistent, thus creating an environment where the contents are more likely to begin to aggregate and become insoluble [19]. Although TDP-43 is one of the most abundant and most aggregate prone constituents of SG [1], this model raises the possibility that the resulting pathological inclusions might also contain TIA1, other SG markers, and other RBP that are typically stored in SG. However, our IHC and IF studies failed to demonstrate any co-localization of TIA1 with TDP-43 in the inclusions, and also failed to show any abnormal accumulation of other RBPs in cases with *TIA1* mutations. Although some other studies have suggested that TIA1 may co-localize with TDP-43 in ALS [17, 32], others have refuted this finding [4]. Consistent with our immunostaining results are previous biochemical studies that failed to show any enrichment of TIA1 in the insoluble protein fraction extracted from post mortem brain tissue of *TIA1* mutation carriers [19]. This suggests that even if TDP-43 begins to form insoluble aggregates in SG, further protein aggregation may occur independent of the presence and function of TIA1. None-the-less, it is possible that the commercial TIA1 antibodies we used are not sufficiently sensitive for use in post mortem tissue that has undergone prolonged fixation and that further TIA1/TDP-43 co-localization studies are warranted.

## Conclusions

In summary, this analysis of a small series of cases provides an initial characterization of the clinical and pathological features in patients with ALS ± FTD caused by mutations in *TIA1*. The clinical presentation is distinguished by frequent bulbar onset weakness and a predominance of expressive aphasia, and by the absence of associated parkinsonian or psychotic features. Although the disease appears to be inherited in an autosomal dominant fashion, the lack of family history in many cases suggests that penetrance may be variable. The striking predominance of females in our series needs to be confirmed, but suggests that expression of the mutation may be influenced by some sex-related factor. The anatomical pattern of neurodegeneration correlates with the main clinical features and these cases tend not to show the caudate atrophy or hippocampal sclerosis that is common with the *C9orf72* mutation. The molecular neuropathology is a combination of FTLD-TDP type B and ALS with TDP-ir inclusions. However, the most striking pathological feature is the consistent presence of round, often LBL inclusions that are visible on HE stain and correlate with an increased frequency of compact TDP-ir NCI in LMN. There is currently no evidence for any abnormal distribution or accumulation of TIA1 protein in postmortem brain tissue of *TIA1* mutation carriers; however, this warrants more detailed investigation. Additional studies are clearly needed to confirm and expand upon this initial characterization. In particular, the presence of *TIA1* mutations needs to be investigated in patients with clinically pure FTD and families with *TIA1* mutations should be reviewed for evidence of multisystemic features such as muscle and bone disease. Even though this initial case series is relatively small, the detailed clinical and pathological characterization will be helpful in identifying additional cases that might carry a *TIA1* mutation and provides a baseline upon which future studies can build.
